# The effectiveness of permethrin-treated deer stations for control of the Lyme disease vector *Ixodes scapularis* on Cape Cod and the islands: a five-year experiment

**DOI:** 10.1186/1756-3305-7-292

**Published:** 2014-06-25

**Authors:** Jason S Grear, Robert Koethe, Bart Hoskins, Robert Hillger, Larry Dapsis, Montira Pongsiri

**Affiliations:** 1 U.S. Environmental Protection Agency, Office of Research and Development, 27 Tarzwell Dr., Narragansett, RI 02882, USA; 2 U.S. Environmental Protection Agency, New England (Region 1), 5 Post Office Square - Suite 100, Boston, MA 02109, USA; 3 Cape Cod Cooperative Extension Service, Barnstable, MA 02630, USA; 4 U.S. Environmental Protection Agency, Office of Research and Development, 1200 Pennsylvania Avenue, N. W., Mail Code 8105R, Washington, DC 20450, USA

**Keywords:** *Ixodes scapularis*, Tick, Permethrin, 4-poster, Feeding station, Host-targeted control, Lyme disease, *Borrelia burgdorferi*, *Odocoileus virginianus*, White-tailed deer, Blacklegged tick

## Abstract

**Background:**

The use of animal host-targeted pesticide application to control blacklegged ticks, which transmit the Lyme disease bacterium between wildlife hosts and humans, is receiving increased attention as an approach to Lyme disease risk management. Included among the attractive features of host-targeted approaches is the reduced need for broad-scale pesticide usage. In the eastern USA, one of the best-known of these approaches is the corn-baited “4-poster” deer feeding station, so named because of the four pesticide-treated rollers that surround the bait troughs. Wildlife visitors to these devices receive an automatic topical application of acaricide, which kills attached ticks before they can reproduce. We conducted a 5-year controlled experiment to estimate the effects of 4-poster stations on tick populations in southeastern Massachusetts, where the incidence of Lyme disease is among the highest in the USA.

**Methods:**

We deployed a total of forty-two 4-posters among seven treatment sites and sampled for nymph and adult ticks at these sites and at seven untreated control sites during each year of the study. Study sites were distributed among Cape Cod, Martha’s Vineyard, and Nantucket. The density of 4-poster deployment was lower than in previous 4-poster studies and resembled or possibly exceeded the levels of effort considered by county experts to be feasible for Lyme disease risk managers.

**Results:**

Relative to controls, blacklegged tick abundance at treated sites was reduced by approximately 8.4%, which is considerably less than in previous 4-poster studies.

**Conclusions:**

In addition to the longer duration and greater replication in our study compared to others, possible but still incomplete explanations for the smaller impact we observed include the lower density of 4-poster deployment as well as landscape and mammalian community characteristics that may complicate the ecological relationship between white-tailed deer and blacklegged tick populations.

## Background

Blacklegged ticks (*Ixodes scapularis*) are the primary vector of Lyme disease between wildlife and human populations in eastern North America, so their abundance during periods of outdoor human activity is a key determinant of Lyme disease risk [1]. Methods to control this abundance are the focus of this study. Another key determinant, which we do not address, is the proportion of these ticks that are infected with the Lyme disease bacterium, *Borrelia burgdorferi.* The biology of this spirochete and the multi-host two year life cycle of blacklegged ticks have produced a highly complex ecological system that continues to challenge ecologists, public health experts, natural resource managers, integrated pest management (IPM) practitioners, and land use planners. Risk management solutions are in various stages of development, some of which require changes in land use practices or the use of biocontrol agents or pesticides that may be harmful to non-target organisms. However, because of the complexity of the Lyme disease ecological system [2] and the limitations and potentially negative impacts of sole reliance on any single available method, it is likely that successful control strategies will require judicious application of an integrated approach consisting of multiple tactics. This necessitates knowledge about the efficacy of specific techniques in varying ecological settings.

White-tailed deer (*Odocoileus virginianus*) are important hosts for adult blacklegged ticks seeking bloodmeals, so their overabundance in the eastern US was historically assumed to be a significant determinant of Lyme disease risk [3]. Massachusetts, like other northeastern states, has seen dramatic increases in white-tailed deer populations. The Massachusetts Audubon Society estimates that fewer than 1000 white-tailed deer existed in the state in 1900; the current estimate is 90,000 (~4.5 km^-2^) [4]. Extirpation of natural predators and increases in forage associated with forest clearing are considered the primary long-term drivers of deer overabundance, with restrictions on hunting in developed areas playing an increasingly important role (reviewed in [5]). However, there is little consensus on the feasibility or effectiveness of specific management techniques for deer population control [6]. Moreover, the mandates of private organizations and local, state, and federal managers of deer and their habitats frequently conflict in ways that complicate coordination [7]. These challenges are exacerbated by the considerable uncertainty about the impact of deer abundance on Lyme disease risk (reviewed in [2]).

As an alternative to direct population control of white-tailed deer, the use of deer-targeted pesticide application via “4-poster” feeding stations to control tick populations is now included among the risk management techniques being tested and in some cases implemented in areas of high Lyme disease incidence [8]. Because of their intended host specificity, 4-posters have the potential to reduce Lyme disease incidence as well as to reduce reliance on residential practices such as broad-spectrum acaricide application. To address the keen interest in quantifying 4-poster effectiveness, we conducted a 5-year controlled study of their effects on blacklegged tick populations on Cape Cod, Nantucket, and Martha’s Vineyard, all of which are in coastal Massachusetts.

White-tailed deer frequently carry heavy burdens of adult stage blacklegged ticks seeking their final blood meal. However, blacklegged ticks have a complex life cycle involving multiple hosts (reviewed in [2]). After feeding to repletion, mated female ticks overwinter and deposit their eggs in the spring. On Cape Cod, deposited eggs typically hatch into larvae in late July and early August and then seek their first blood meal. If this search results in a blood meal from a host infected with the Lyme disease bacterium, *Borrelia burgdorferi,* and if transmission occurs, then the larva becomes infected. After feeding to repletion, larvae moult into nymphs. After overwintering, each nymph seeks a new host for what is typically the second blood meal in its life cycle. This second host is an additional opportunity for the tick to acquire the Lyme disease spirochete. In late summer, these nymphs moult into the adult stage and seek their final blood meal. All stages of feeding ticks are potentially affected by exposure of their hosts to 4-poster treatments, but this exposure is expected to be highest for adult ticks because of their relatively high abundance on large vertebrates. The impacts of 4-posters include direct mortality to larvae or nymphs attached to 4-poster visitors and reduced numbers of eggs due to reductions in adult populations. Our study was designed to estimate the magnitude of these effects by repeated sampling of nymph and adult ticks at 4-poster sites and untreated control sites.

Several previous studies have reported large reductions in tick abundance in areas treated with 4-posters relative to untreated control areas. Most notably, a coordinated six-year study in the northeastern US reported approximately 70% reduction in nymphs at the end of the study [9-13]. Only one of the five separately published studies contained independent within-site replication, so meta-analysis of the five sites became an important basis for inference about 4-poster effectiveness. Although the meta-analysis by Brei *et al*. [12] appears to have treated multiple samples from each site as statistically independent samples, the results at the northeast regional scale are compelling.

The northeast regional study deployed > 100 4-posters across its five study areas at a density of 4 to 5 stations km^-2^ (0.016 stations acre^-1^). We were interested in estimating 4-poster efficacy for coastal southeastern Massachusetts and used a single controlled experiment with site replication. We expected our study to produce a geographically narrower but more statistically robust confirmation of the broader regional findings reported by Pound *et al.*[11]. In addition, we sought to refine design considerations for longer term deployment of 4-poster devices in southeastern Massachusetts. Given the rapid and dramatic effects seen in previous studies, we anticipated that 4-poster deployment at 1–2 stations km^-2^ (< 0.007 stations acre^-1^), or approximately 40% of the density used in the northeast regional study, would produce measurable effects at a more feasible deployment density for area resource managers.

We conducted our 4-poster study in southeastern Massachusetts, where Lyme disease poses a serious health risk. Massachusetts ranks among the top 10 states in Lyme disease annual reporting to the US Centers for Disease Control [14]. In recent reporting, these top 10 states accounted for more than 93% of the total cases reported nationally over the 15 yr period documented in the report. Two counties in the region of southeastern Massachusetts where our study was conducted were among the top 10 counties nationally for average rate of Lyme disease incidence (reported cases) during the period 1997–2006 [14]. Our study was motivated by these factors and the need for environmentally sustainable management practices for reducing Lyme disease risk.

## Methods

Deer 4-poster stations were activated in the fall of 2007 (mid August to mid November) and in spring (mid March to mid June) and fall of all subsequent years (2008–2011) at precisely the same locations each year (within 2 m of initial locations). Closure of stations during winter was partly the result of regulations prohibiting wildlife provisioning during the hunting season. At each site, multiple stations were distributed at approximately one station per 150 acres (1.65 stations km^-2^), based in part on results from previous studies [15].

**Figure 1:**
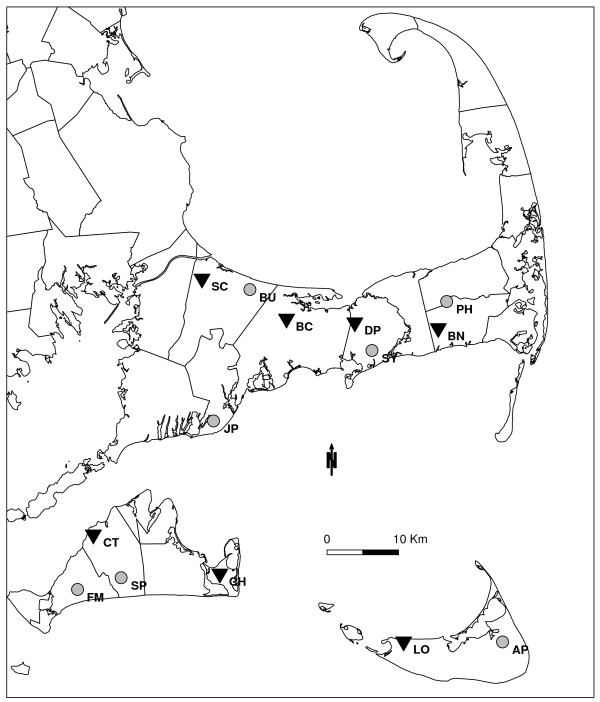
**Locations of treated sites (triangles) and controls (filled circles) in the 5-year study of 4-poster deer feeding station effects on blacklegged tick abundance.** Treated sites had multiple 4-poster stations; all sites had multiple tick drag sampling locations. Site abbreviations are: SC = Shawme Crowell; BU = Burgess; BC = Bridge Creek; DP = Dennis Pond; SY = Syrjala; PH = Punk Horn; BN = Bell’s Neck; JP = Jehu Pond; FM = Fulling Mill; CT = Cedar Tree Neck; SP = Sepiessa Point; CH = Chappaquiddick; LO = Loring Nature Center; and AP = Almanack Pond.

Selection of sites for this study was based on: 1) history of an active blacklegged tick population; 2) evidence of white tailed deer; 3) accessibility for maintenance and input of corn bait and permethrin; and 4) distance from residences (> 91 m). This resulted in seven treatment sites on Nantucket, Martha’s Vineyard and Cape Cod (Figure 1). Comparable control sites (i.e., without 4-poster stations) were chosen based on location (> 1.6 km from treated sites), habitat, and presence of blacklegged ticks. This relatively low density of sites and of 4-poster stations within these sites (1–2 stations km^-2^) was considered indicative of what tick control programs can realistically be expected to maintain in the study area.

During periods of activation, each station was maintained weekly or biweekly, with corn added *ad libidum* and permethrin acaricide added to rollers at a rate of 7.5 ml per 50 lbs (23 kg) of corn consumed. Inputs to each station, including the amount of corn consumed monthly, the amount of permethrin added, the number of station visits, as well as any necessary replacements or repairs were recorded. Beginning in spring 2007 (before station deployment), nymph ticks were sampled at all treatment and control sites in May, June and July of each year using a cloth dragging procedure [16] whereby a 0.46 m^2^ (50.8 × 90.4 cm) double-sided white flannel cloth was dragged along the ground at the edge of a trail or wooded road for 30 seconds at approximately one yard per second. This procedure was repeated along fixed transects in October and November of each year for collection of adult ticks. This resulted in a total of 9890 drags approximately evenly distributed across sites (Table 1). Thus, each site was sampled 4–5 times between 1 May and 10 Nov of each year, for a total of approximately 24 sampling events (30 drags per visit per site for each site over the study period; see Table 1 for deviations). This is a relatively high sampling frequency and was intended to overcome under-sampling problems [17].

**Table 1:** Number of tick drag samples by treatment, site, and year for Cape Cod, Martha’s Vineyard and Nantucket

			**No. of**	**Number of drag samples**				
		**Site**	**4-Posters**	**2007**	**2008**	**2009**	**2010**	**2011**
Cape cod	Control	Burgess	-	150	170	130	180	180
		Jehu Pond	-	60	180	90	150	180
		Punk Horn	-	60	180	90	180	180
		Syrjala	-	150	150	90	180	180
	Treated	Bells Neck	4	120	180	90	180	180
		Bridge Creek	4	150	150	60	180	180
		Dennis Pond	4	150	180	90	150	180
		Shawme Crowell	6	150	180	90	180	180
Martha's vineyard	Control	Fulling Mill	-	80	180	90	150	180
		Sepiessa Point	-	60	180	90	150	180
	Treated	Cedar Tree Neck	15	90	180	90	150	180
		Chappaquiddick	5	90	180	90	150	180
Nantucket	Control	Almanack Pond	-	30	140	90	160	180
	Treated	Loring Nature Center	4	60	150	90	180	180

**Figure 2:**
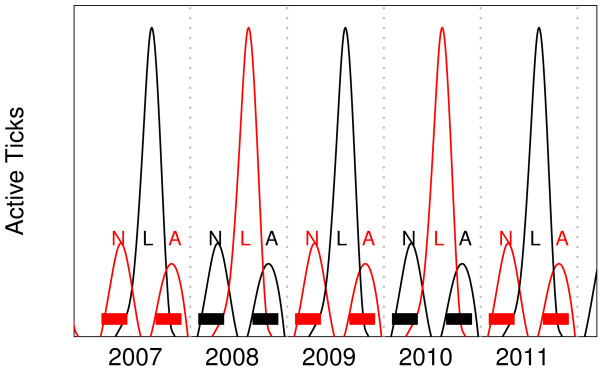
**Diagram of sampling schedule (filled rectangles) superimposed on expected abundances of active ticks, *Ixodes scapularis*), which breeds only at the end of its two-year life cycle.** Two overlapping populations are present at any given time and are represented here as different shades. N, L, and A denote periods of nymph, larval, and adult activity. Relative abundances are based on Figure eight in Ostfeld [2].

For statistical analyses and prediction, we used log-linear negative binomial models with random effects (GLMM; generalized linear mixed effects models). Life stage, treatment, and time were treated as fixed effects. Each statistical formulation was fitted using either days or years elapsed since the beginning of the study. Each of the 42 transects in the study was assigned a unique ID and treated as a random effect. The random effects were modeled as effects on intercepts only and were included because of the expected correlation between repeated samples taken from each transect over the course of the study. This is intended to address microclimate or other unknown but persistent differences between sites. The negative binomial distribution was used because of the high variance to mean ratio in the data, as is common in tick sampling data due to patchy spatial distribution (see [17] for analysis of sampling implications). Because of the two-year semelparous life cycle, nymphs and adults sampled in a given year are predominantly descendents of nymphs and adults sampled two years earlier. Thus, the longest time series for a given population in our study is represented by samples from 2007, 2009 and 2011 (Figure 2). Our statistical analyses focused on these samples.

These log-linear models were used to evaluate statistical evidence for 4-poster treatment effects on nymphal and adult tick abundances and to estimate the size of these effects. Each of the candidate statistical models represented a specific hypothesized explanation of the data. Thus, the set included a ‘no effects’ model, a ‘treatment only’ model, a ‘time only’ model, a ‘treatment + time’ model, and a ‘treatment × time’ model. Evidence for 4-poster effects would be indicated by strong statistical support for models containing treatment effects. Support for a ‘time only’ model would indicate a regional change in tick abundance unrelated to 4-poster effects. Each model was fitted as a GLMM using the R implementation of AD Model Builder [18,19]. Support for each model was assessed using corrected Akaike Information Criteria (AICc; see Section 2.2 in [20]). AIC*c* weights were used to compute model-weighted predictions of tick density and unconditional standard errors for 95% confidence limits (eqn 6.12 in [20]). This so-called information-theoretic approach enables fuller extraction of the information contained in the data and allows evidence-based ranking of candidate models. When multiple models are supported (i.e., knowledge of the study system is uncertain), the final estimate of effect size (i.e., 4-poster effect) and its confidence limits incorporate the influence of all supported models. For this reason, the rejection of models via *p*-value cutoffs does not arise in our analysis.

We used Abbott’s formula [21] to compute percent reduction of ticks relative to controls for comparison to other studies e.g., [13]. Specifically,

$$\text{Pct}\;\text{Control}\;=\;100\;\times \left(1-\frac{E_{0,\mathrm{ctrl}}\times E_{t,\mathrm{trt}}}{E_{t,\mathrm{ctrl}}\times E_{0,\mathrm{trt}}}\right)$$

represents the effect of treatments between time *t* = 0 and *t* = t, where *E* denotes mean abundance at control (cntrl) and treated (trt) sites predicted from the statistical models.

Pelage swab samples from white-tailed deer carcasses were collected prior to meat processing at hunter check-in stations on Chappaquiddick Island, Edgartown, MA, which we assume supports a closed deer population (no immigration or emigration). These samples were collected by wiping a cotton gauze pad on the neck, throat and chin area of each deer for thirty seconds. The samples were placed in amber glass vials and stored frozen. Samples were shipped on ice by overnight delivery to the Massachusetts Pesticide Analysis Laboratory for permethrin residue analysis using hexane extraction followed by gas chromatography with electron capture detection and mass spectrometry. Data from island hunters were used with these residue analyses to estimate the proportion of deer treated topically within the treatment zone.

## Results

**Figure 3:**
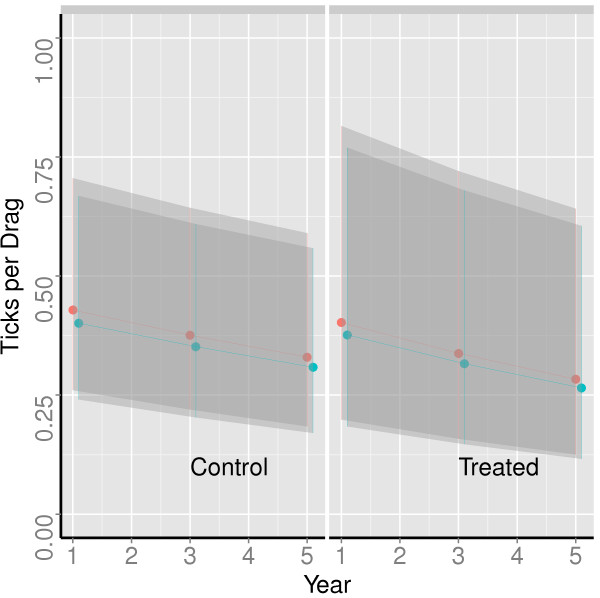
**Predicted counts and 95% confidence limits (shaded areas) for blacklegged tick nymphs (red) and adults (blue) during 30-second drags on June 1 (nymphs) and October 1 (adults) during years 1, 3, and 5 of the study.** Predictions are computed using AIC*c*-weighted averaging of all candidate log-linear generalized mixed effects models, but standard errors used for confidence intervals are based on fixed effect variance only.

**Figure 4:**
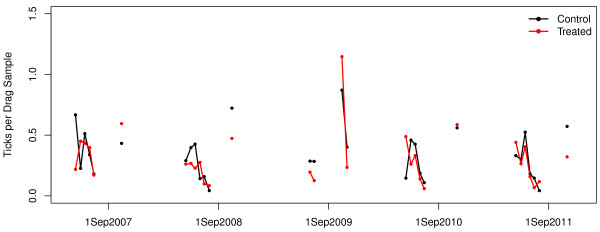
**Graphical summary of tick drag data aggregated into means for 15-day intervals.** Each point is the mean of all drags within the 15-day window for all 7 sites of the given treatment level. Average number of drags for each point is 330. See Methods section and Table 1 for additional details on distribution of sampling effort.

The model containing interactions between 4-poster treatment and time was the best fitting (based on log likelihood) and most parsimonious (based on AIC*c*) of the models we used to analyze tick sampling data (Table 2). The interaction term in this model is interpreted as evidence that the treatments caused a stronger tick decline than was observed at the control sites. However, there was modest support in the data for two models without the interaction (i.e., ΔAIC*c* < 2; Table 2), leading to model selection uncertainty [20]. As a result of this uncertainty, we used AIC*c*-weighted model averaging to make predictions about treatment effects on tick abundance (Figure 3).Using Abbott’s formula with the model-averaged estimates of treatment effects, our estimate of Pct Control was 8.4%, which is substantially lower than that reported for other studies. This estimate increases to 20% when only the interaction model is used by itself (rather than model-averaged estimates), but as already noted, inference based solely on this model is not supported by our data. Visual representation of aggregated drag counts (Figure 4) is consistent with the small effect detected in our statistical analyses.

**Table 2:** **AIC statistics for models of tick treatment effects on tick drag sample abundances**^**1**^

**Model (fixed effects)**	**Log likelihood**	***K***^**2**^	**ΔAIC** ***c***	**AIC** ***c*** **weight**
Stage + Trt × Year	-7757.3	8	0.0	0.40
Stage + Year	-7759.3	6	0.1	0.39
Stage + Trt + Year	-7758.9	7	1.3	0.21
Stage + Trt × Day	-7761.8	8	9.0	0.00
Stage + Day	-7764.1	6	9.7	0.00
Stage + Trt + Day	-7763.7	7	10.9	0.00
Stage	-7772.3	5	24.1	0.00
Stage + Trt	-7772.0	6	25.5	0.00

^1^Data are from the 2007, 2009 and 2011 cohort of blacklegged ticks in a 4-poster study in southeastern Massachusetts. See Section 2.2 of Burnham and Anderson [20] for an explanation of AIC statistics.

^2^*k* is number of parameters in the model.

The rates of pesticide residue detections on pelage swabs (gauze pads; detection limit = 0.02 ug residue pad^-1^) collected from harvested deer on Chappaquiddick Island were 0.12, 0.69, 0.47, and 0.7 detections per deer for 2007, 2008, 2010, and 2011, respectively. The low number for 2007 and low rate of corn replenishment during station maintenance in that year suggest the possibility of a period of low deer usage during initial habitation to the station locations.

## Discussion

We detected a relatively modest effect of 4-posters on blacklegged tick abundances in our coastal Massachusetts study area. Thus, our experiment supports previous findings that 4-posters reduce tick abundance, but the effect size we observed was smaller (Figure 3). Our study is the first to our knowledge that combines: 1) sampling over multiple generations and across multiple control and treatment replicates; 2) analysis of all nymph and adult tick data for a cohort population in a single count-based statistical model; and 3) detailed treatment of the repeated measures sampling design. The importance of these analytical considerations is described by Carroll *et al*. [9]. Although they dissected their analysis into separate comparisons between pairs of years, differences in effect size between our results, those of Carroll *et al.*[9] and other findings from the USDA Northeast Regional Study [11], are probably not due solely to differences in statistical methods or levels of replication. Uncertainty was also larger in our study compared with the meta-analytic results of Brei *et al.*[12], perhaps because we addressed model selection uncertainty and did not treat co-located stations or transects as statistically independent samples. Large deer home range size may reduce statistical independence of our study sites, but is considered less than 1.6 km in radius within seasons and possibly decreases in areas of high deer density (reviewed in [22]; also see [23]). Distances between our treated and control sites were always at least twice this radius, but note that any violation of the independence assumption would mean that our uncertainty estimate (i.e., the width of confidence limits in Figure 3) is too low and differences between our results and those of the USDA study may be even larger than what we have reported here. However, despite these potentially important analytical differences, we suspect that most of the difference between our results and those of others is due to the wider spacing of our 4-poster devices (1–2 stations km^-2^ vs. 4–5 stations km^-2^ in the USDA study). Other differences between studies may include deer densities and the operational periods for which the stations were maintained. Also, our study used permethrin as the acaricidal ingredient whereas the USDA study used amitraz. We are unaware of any known differences in effectiveness of these ingredients when used in 4-posters, but permethrin has been shown to be considerably more toxic than amitraz to several species of *Amblyomma* ticks [24,25].

In the region of our study, the Commonwealth of Massachusetts seeks to manage white-tailed deer abundances at a density of 6–8 deer mi^-2^ (2.8 – 3.1 deer km^-2^), primarily through recreational hunting allowances [4]. However, significant variation in deer abundance likely exists among our three study areas (Cape Cod, Martha’s Vineyard and Nantucket). Although our study was not designed to detect differences in 4-poster deer visitation among these areas, average annual corn consumption differed considerably based on rates of 4-poster replenishment (81, 182, and 326 kg station^-1^ yr^-1^ for Cape Cod, Martha’s Vineyard and Nantucket, respectively). Since station density was similar across sites, these consumption rates should be roughly indicative of deer density if relative corn consumption by non-target species is also similar across sites. Indeed, the State of Massachusetts estimates deer densities on Cape Cod to be much closer to its management goal than on the islands, where densities may be more than 15 deer km^-2^[26].

Experimental exclusion of deer has been shown to affect the density of blacklegged ticks [27,28] (but see [29]), but the effects of these and other deer control experiments on human disease risk are not clear [2,30]. This is partly because deer are ineffective hosts of Lyme disease – Telford *et al.*[31] reported that only about 1% of ticks became infected after feeding on deer – and thus, as members of a larger host community, may contribute to a dilution effect on infection prevalence among questing ticks (demonstrated theoretically in [32]; empirical evidence for dilution in other disease systems is reviewed in [33]). If the role of deer in supporting tick populations is as large as commonly believed, successful management of tick abundance through technologies such as the 4-poster device could reduce the assumed need for deer eradication. However, the number of surviving, untreated deer that would be sufficient to support high tick abundance is difficult to estimate. The highest per capita deer treatment rate observed in our pelage residue samples from Chappaquiddick was 70%. Since the frequency distribution of ticks on deer is poorly known, it is possible that only a few untreated deer could weaken 4-poster effects. If such incomplete herd treatment does occur, social exclusion of subdominant individuals from feeding stations may also be important to consider (*personal communication*, M. Maquire, Cape Cod Cooperative Extension). These complexities, the existence of alternative tick hosts that might support tick abundance in the absence of deer or compensate for high mortality on treated deer, and the unknown degree to which these other hosts visit the 4-posters are all potentially important factors in the interpretation of tick abundance data such as ours.

Since there is no currently available pharmacological solution to Lyme disease, risk management focuses on reducing the likelihood of tick bites. The suite of management techniques includes modification of landscapes to reduce habitat suitability for ticks and their hosts, hunting programs to control deer populations, application of pesticides to the landscape, application of pesticides targeted to potential hosts (e.g., 4-posters) and increase of human awareness to modify behavior and promote personal protection practices. Some of these methods have been shown to affect the Lyme disease ecology (and presumably risk), but to varying degrees that depend on the ecological context, scale, and other details of the application. Landscape-scale experimental and observation programs that incorporate ecological and epidemiological approaches would help to identify those critical contextual details that should inform the balance of techniques. At that point, holistic and sustainable risk management strategies would be within reach.

## Conclusions

The relatively modest effect of 4-posters on tick abundance in this five-year experiment, compared to larger effects seen in other studies, can possibly be explained by landscape characteristics, deer density and vertebrate host community composition in our study area, and the density of 4-poster stations we deployed. An important management implication is that the role of deer in the Lyme disease system may be more complicated than previously expected. It is important to weigh this possibility against concerns from the wildlife management community about the effects of wildlife provisioning and increased social contact between wildlife visitors at the 4-poster stations (e.g., wildlife disease transmission). This means that 4-posters deserve further study, experimental application, and refinement, but do not represent a low cost ‘silver bullet’ in the control of Lyme disease except perhaps under specific circumstances that remain to be identified. This is unsurprising given the complexity of the Lyme disease ecological system. 4-posters should be considered part of a broader suite of strategies, the most sustainable of which in the long term will embrace the strong linkages between ecological health and human disease risk and will support the differing mandates of environmental stewardship, wildlife management, and public health organizations.

## Competing interests

The authors declare that they have no competing interests.

## Authors’ contributions

All authors made substantial contributions to conception and design of this study and the interpretation of results. In addition, RK, BH and RH contributed to the review and oversight of field research. LD carried out and/or supervised the 4-poster station maintenance and tick and deer residue sampling. JG designed and performed the statistical analyses and modeling, prepared the manuscript and coordinated its review and revision by the participating authors, all of whom read and approved the final version.

## References

[B1] Barbour AG Fish D The biological and social phenomenon of Lyme disease Science 1993 260 1610 1616 8503006 10.1126/science.8503006

[B2] Ostfeld RS Lyme disease: The ecology of a complex system 2011 New York, NY: Oxford University Press

[B3] Spielman A Wilson ML Levine JF Piesman J Ecology of *Ixodes dammini*-borne human babesiosis and Lyme disease Annu Rev Entomol 1985 30 439 460 3882050 10.1146/annurev.en.30.010185.002255

[B4] Massachusetts Department of Fish and Game Massachusettes fish and wildlife guide to hunting, freshwater fishing and trapping 2011 Williamstown, MA: JF Griffin Publishing

[B5] Cote SD Rooney TP Tremblay J-P Dussault C Waller DM Ecological impacts of deer overabundance Annu Rev Ecol Evol Syst 2004 35 113 147

[B6] Brown TL Decker DJ Riley SJ Enck JW Lauber TB The future of hunting as a mechanism to control white-tailed deer populations Wildl Soc Bull 2000 28 797 807

[B7] Porter WF Underwood HB Of elephants and blind men: Deer management in the U.S. national parks Ecol Appl 1999 9 3 9

[B8] Pound JM Miller JA George JE LeMeilleur CA The "4-poster" passive topical treatment device to apply acaricide for controlling ticks (Acari: Ixodidae) feeding on white-tailed deer J Med Entomol 2000 37 588 594 10916301 10.1603/0022-2585-37.4.588

[B9] Carroll JF Hill DE Allen PC Young KW Miramontes E Kramer M Pound JM Miller JA George JE The impact of 4-poster deer self-treatment devices at three locations in Maryland Vector Borne Zoonotic Dis 2009 9 407 U464 19650735 10.1089/vbz.2008.0165

[B10] Daniels TJ Falco RC McHugh EE Vellozzi J Boccia T Denicola AJ Pound JM Miller JA George JE Fish D Acaricidal treatment of white-tailed deer to control ixodes scapularis (Acari: Ixodidae) in a New York Lyme disease-endemic community Vector Borne Zoonotic Dis 2009 9 381 387 19650732 10.1089/vbz.2008.0197

[B11] Pound JM Miller JA George JE Fish D Carroll JF Schulze TL Daniels TJ Falco RC Stafford KC III Mather TN The United States department of agriculture’s Northeast area-wide tick control project: summary and conclusions Vector Borne Zoonotic Dis 2009 9 439 448 19650739 10.1089/vbz.2008.0200

[B12] Brei B Brownstein JS George JE Pound JM Miller JA Daniels TJ Falco RC Stafford KC III Schulze TL Mather TN Carroll JF Fish D Evaluation of the United States department of agriculture Northeast area-wide tick control project by meta-analysis Vector Borne Zoonotic Dis 2009 9 423 430 19650737 10.1089/vbz.2008.0150 PMC2904192

[B13] Stafford KC III Denicola AJ Pound JM Miller JA George JE Topical treatment of white-tailed deer with an acaricide for the control of ***Ixodes scapularis*** (Acari: Ixodidae) in a connecticut lyme borreliosis hyperendemic community Vector Borne Zoonotic Dis 2009 9 371 379 19650731 10.1089/vbz.2008.0161

[B14] CDC Morbidity and mortality weekly report: surveillance for lyme disease - United States, 1992–2006 57 http://www.r-project.org/: Centers for Disease Control; 2008 18830214

[B15] Williams SC DeNicola AJ Brittingham MC, Kays J, McPeake R Spatial movements in response to baiting female whitetailed deer Proceedings of the 9 th Wildlife Damage Management Conference; 5–8 October 2000 2000 State College, PA 206 224

[B16] Falco RC Fish D A comparison of methods for sampling the deer tick, *Ixodes dammini*, in a Lyme disease endemic area Exp Appl Acarol 1992 14 165 173 1638929 10.1007/BF01219108

[B17] Dobson AD Ticks in the wrong boxes: assessing error in blanket-drag studies due to occasional sampling Parasit Vectors 2013 6 344 24321224 10.1186/1756-3305-6-344 PMC4029458

[B18] Fournier DA Skaug HJ Ancheta J Ianelli J Magnusson A Maunder MN Nielsen A Sibert J AD Model Builder: using automatic differentiation for statistical inference of highly parameterized complex nonlinear models Optim Methods Softw 2012 27 233 249

[B19] R Core Team R: A Language and Environment for Statistical Computing 2012 2.15.0 Vienna, Austria: R Foundation for Statistical Computing

[B20] Burnham KP Anderson DR Model selection and multimodel inference 2002 New York, NY: Springer

[B21] Abbott WS A method for computing the effectiveness of an insecticide J Econ Entomol 1925 18 265 267

[B22] Smith WP Odocoileus virginianus Mammalian Species 1991 388 1 13

[B23] Tierson WC Mattfeld GF Richard W Sage J Behrend DF Seasonal movements and home ranges of white-tailed deer in the Adirondacks J Wildl Manage 1985 49 760 769

[B24] Burridge MJ Peter TF Allan SA Mahan SM Evaluation of safety and efficacy of acaricides for control of the african tortoise tick (*Amblyomma marmoreum*) on leopard tortoises (*Geochelone pardalis*) J Zoo Wildl Med 2002 33 52 57 12216793 10.1638/1042-7260(2002)033[0052:EOSAEO]2.0.CO;2

[B25] Burridge MJ Simmons L-A Allan SA Efficacy of acaricides for control of four tick species of agricultural and public health significance in the United States J Agric Urban Entomol 2003 20 207 219

[B26] Massachusetts Division of Fish and Wildlife Deer management overview State of Massachusetts; 2014. http://www.mass.gov/eea/agencies/dfg/dfw/fish-wildlife-plants/mammals/deer-management.html.

[B27] Stafford KC III Reduced abundance of ***Ixodes scapularis*** (Acari: Ixodidae) with exclusion of deer by electric fencing J Med Entomol 1993 20 986 996 8271257 10.1093/jmedent/30.6.986

[B28] Wilson ML Telford SR III Piesman J Spielman A Reduced abundance of *Ixodes dammini* (Acari: Ixodidae) following elimination of deer J Med Entomol 1988 25 224 228 3404540 10.1093/jmedent/25.4.224

[B29] Wilson ML Levine JF Spielman A Effect of deer reduction on abundance of the deer tick (*Ixodes dammini*) Yale J Biol Med 1984 57 697 705 6516462 PMC2589992

[B30] Perkins SE Cattadori IM Tagliapietra V Rizzoli AP Hudson PJ Localized deer absence leads to tick amplification Ecology 2006 87 1981 1986 16937637 10.1890/0012-9658(2006)87[1981:ldaltt]2.0.co;2

[B31] Telford SR III Mather TN Moore SIW Wilson ML Spielman A Incompetence of deer as reservoirs of the Lyme disease spirochete Am J Trop Med Hyg 1988 39 105 109 3400797 10.4269/ajtmh.1988.39.105

[B32] VanBuskirk J Ostfeld RS Controlling Lyme disease by modifying the density and species composition of tick hosts Ecol Appl 1995 5 1133 1140

[B33] Johnson PTJ Thieltges DW Diversity, decoys and the dilution effect: how ecological communities affect disease risk Journal of Exp Biol 2010 213 961 970 20190121 10.1242/jeb.037721

